# Aortopathy associated with bicuspid aortic valve: advances in clinical and hemodynamics research

**DOI:** 10.3389/fphys.2025.1576072

**Published:** 2025-05-06

**Authors:** Minghui Yang, Zixiong Nie, Honghua Yue, Weitao Liang, Zhong Wu

**Affiliations:** Department of Cardiovascular Surgery, West China Hospital, Sichuan University, Chengdu, China

**Keywords:** bicuspid aortic valve, WSS, aortopathy, hemodynamics, aortic dilation, paediatric BAV

## Abstract

Having a bicuspid aortic valve (BAV) is the most common congenital heart disease, affecting 0.5%–2% of the population, with significant heterogeneity in clinical presentation, complications, and outcomes. Hemodynamic disturbances, including wall shear stress (WSS), eccentric flow, helical flow and energy turbulence, are critical in the development and progression of BAV-associated aortopathy, which is characterized by ascending aortic dilation, aortic aneurysm, and dissection. The interplay between genetic factors and hemodynamic abnormalities further complicates disease mechanisms, influencing clinical management and prognosis. To investigate the hemodynamic characteristics of BAV-associated aortic disease before and after surgery, this study reviewed recent advances in the understanding of the hemodynamic and genetic mechanisms underlying BAV-associated aortic disease, as well as clinical treatment strategies and recommendations for managing cases with additional genetic factors. This paper systematically summarizes the changes in hemodynamic parameters related to aortopathy in patients with BAV before and after surgery and their correlation with aortic dilation. This paper also explores the influence of different aortic valve morphotypes and functional phenotypes on hemodynamic parameters. Notably, this review focuses on the unique hemodynamic features of paediatric and young patients with BAVs and reviews clinical management recommendations for this group. The relationship between postoperative hemodynamic changes and clinical outcomes, such as redilation and long-term survival rates, warrants further exploration in BAV patients.

## 1 Introduction

**FIGURE 1:**
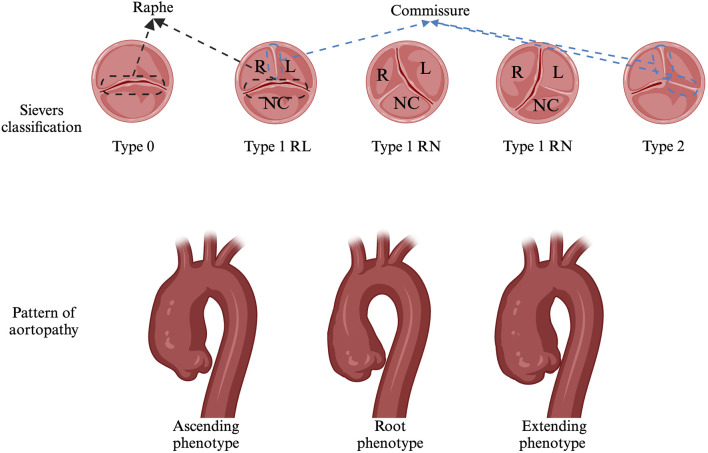
Sievers classification and Pattern of aortopathy..

Having a bicuspid aortic valve (BAV) is the most common congenital heart disease, with a prevalence of 0.5%–2%, and it occurs approximately three times more frequently in males (Michelena et al., 2008; Siu and Silversides, 2010; Kong et al., 2017b). The clinical manifestations, therapeutic approaches, complications, and prognosis of BAV are highly heterogeneous. The Sievers classification (Figure 1) is widely used to characterize BAV valve fusion morphophenotypes by counting raphes and assessing cusp and raphe fusion orientations (Sievers and Schmidtke, 2007). The phenotype-based nomenclature predicts complications and outcomes and guides follow-up strategies. For example, patients with right and noncoronary cusp fusion (RN) exhibit more severe aortic valve dysfunction and require earlier interventions. Patients with RN display greater ascending aortic (AAo) dilation than do those with right and left cusp fusion (RL), while RL is more commonly associated with aortic root dilation (Grattan et al., 2020). BAV manifests variably in children and adolescents, depending on onset age, prompting studies to classify it by clinical phenotype (Niaz et al., 2020). To standardize the terminology, an international consensus in 2021 proposed a new clinical classification system dividing patients with BAVs into three subgroups: (i) Complex valvulo-aortopathy, characterized by more severe clinical and pathological phenotypes, often associated with syndromic conditions. (ii) Typical valvulo-aortopathy, the most common subgroup, which includes progressive BAV dysfunction and/or aortic dilation without significant associated syndromic conditions. (iii) Uncomplicated or undiagnosed BAV, representing a lifelong asymptomatic condition associated with mild or nonprogressive valvular disease, with no distinctive clinical features (Michelena et al., 2021).

Patients with BAVs may present in various clinical scenarios, ranging from asymptomatic young children identified during routine examinations to elderly patients with end-stage disease and severe complications (Michelena et al., 2014; Michelena et al., 2018). Compared with patients with tricuspid aortic valves (TAVs) have a significantly greater cardiovascular risk (Mennander et al., 2020). The most common complications of BAVs are valvular dysfunctions such as aortic stenosis (AS) (14%–50%) and aortic regurgitation (AR) (23%–70%) which affect 50%–86% of cases and represent the primary indications for aortic valve surgery (Roberts and Ko, 2005; Michelena et al., 2011; Detaint et al., 2014; Kong et al., 2017a; Evangelista et al., 2018; Cheng et al., 2021; Yang et al., 2023). Aortic dilation occurs in more than 50% of BAV patients, but its prevalence varies widely across studies due to inconsistent definitions, particularly in paediatric and adolescent populations (Detaint et al., 2014; Evangelista et al., 2018; Yang et al., 2020). Progressive thoracic aortic dilation increases the risk of cardiovascular events such as aortic dissection or thoracic aortic aneurysm rupture, but the absolute incidence is relatively low (<1%) (Fedak et al., 2002; Yang et al., 2020). Additionally, infective endocarditis (∼2%), mitral valve prolapse (1.6%–2.7%), and heart failure (7% ± 2%) are notable complications (Michelena et al., 2008; Padang et al., 2019). In paediatric BAV patients, the primary complications are similar to those in adults (Michelena et al., 2008; Padang et al., 2019). Overall, aortic diameter and moderate-to-severe AS were significantly associated with all-cause mortality (Ye et al., 2023). Therefore, monitoring the diameter and progression of aortopathy is crucial for predicting clinical outcomes and assessing the risks of complications.

**FIGURE 2:**
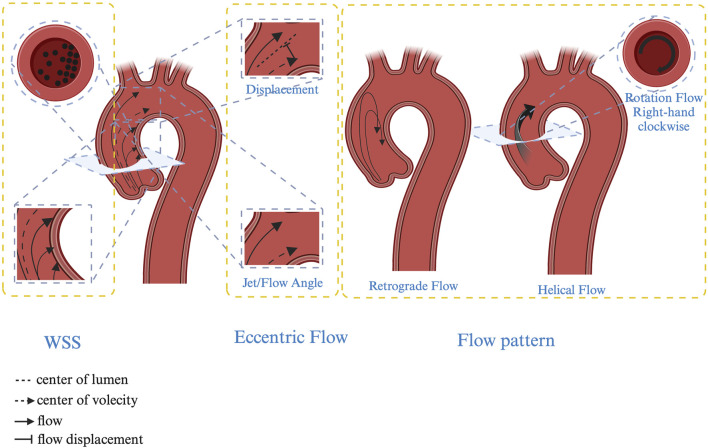
Hemodynamic characteristics in BAV patients. Jet/Flow angle: The angle of the jet relative to the central line; Displacement: The distance between the lumen centre and velocity centre, typically standardized by the lumen diameter. Wall shear stress (WSS): The tangential force per unit area exerted on the aortic wall. Rotational/Helical flow: The integral of vorticity across the cross-sectional area. Retrograde flow: Reverse flow occurring along the longitudinal axis of the lumen during systole.

Transthoracic echocardiography (TTE) and transoesophageal echocardiography are commonly used to diagnose and classify BAV (Sievers and Schmidtke, 2007; Galian-Gay et al., 2020; Cramer and Prakash, 2019). However, relying solely on diameter or volume may not fully represent the progression of aortopathy, necessitating the incorporation of hemodynamic assessments. With advances in technology, new techniques such as ECG-gated CT and MRI angiography for observing valve morphology, 3D or 4D flow parameter reconstruction and artificial intelligence machine learning have been employed to analyse changes in aortic hemodynamics (Franco et al., 2022; Peper et al., 2022). These innovations provide novel predictive parameters for the clinical management and prognosis of aortic complications. Among the extensively studied parameters are the following: Jet angle: The angle of the jet relative to the central line. Normalized displacement: The distance between the lumen centre and velocity centre, typically standardized by the lumen diameter. Wall shear stress (WSS): The tangential force per unit area exerted on the aortic wall. Rotational/Helical flow: The integral of vorticity across the cross-sectional area. Retrograde flow: Reverse flow occurring along the longitudinal axis of the lumen during systole. Turbulent kinetic energy (TKE): The intensity of velocity fluctuations due to turbulence (Figure 2) (Bissell et al., 2023).

**FIGURE 3:**
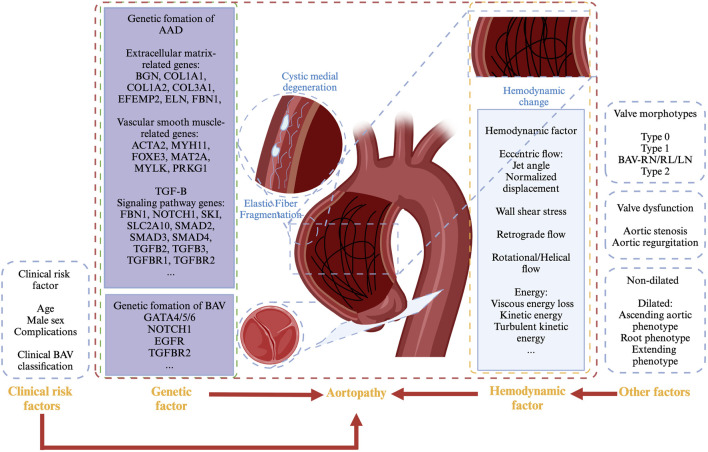
Mechanism of aortopathy in BAV patients.

Aortic dilation typically begins in childhood and progresses gradually, with the incidence of ascending aortic dilation increasing with age. It is currently believed that aortopathy in BAV patients may be caused by genetic defects in the vascular wall, hemodynamic changes, or a combination of both factors, which remains a subject of significant discussion and debate (Figure 3). (Mahadevia et al., 2014; Guala et al., 2022; Minderhoud et al., 2022; Soulat et al., 2022).

## 2 Aortopathy

Aortopathy, including aortic dilation, aortic aneurysm, and aortic dissection, is an ongoing process in BAV patients. The definition of aortic aneurysm in the aortic root and ascending aorta differs from that of aneurysms in other parts of the aorta, where an aneurysm is typically defined as having a diameter greater than 1.5 times the average size. However, aneurysms that occur in these regions are much smaller than this threshold (Johnston et al., 1991; Isselbacher et al., 2022). Many studies suggest that, in comparison with the general population, the risk of aortic aneurysm or dissection begins to rise when the ascending aorta diameter exceeds 40 mm, with a significant increase in risk at 45 mm, particularly for BAV patients (Borger et al., 2004; Paruchuri et al., 2015). Therefore, defining aortic dilation as a diameter greater than 40 mm is reasonable, whereas 45 mm is defined as the threshold for an aortic aneurysm (Isselbacher et al., 2022). The diameter of the aorta is influenced by various factors, including age, sex, body size, genetic predisposition, comorbidities, measurement site, and accuracy (Hiratzka et al., 2010; Devereux et al., 2012; Donadei et al., 2019; Modica et al., 2022; Keuning et al., 2024).

Defects in vascular wall integrity have been implicated as a pathophysiological mechanism in BAV-associated aortopathy. Complex inflammatory reactions and tissue changes, such as fragmentation of elastic fibres, release of matrix metalloproteinases, and structural alterations in vascular smooth muscle cells within the tunica media, may lead to progressive cystic medial necrosis and dysfunction of the aortic media (Nataatmadja et al., 2003; Pisano et al., 2012).

### 2.1 Patterns of aortopathy and relationships with BAV morphotypes

Aortopathy affects all segments of the aorta, from the root to the mid-segment of the proximal aortic arch. In the 2022 guidelines, dilation phenotypes were redefined as the ascending phenotype, extending phenotype, and root phenotype (Figure 1). Ascending phenotype: Features of tubular dilation of the AAo, especially along its curvature, are often accompanied by varying degrees of root dilation, which is associated with increased age at diagnosis (>50 years) and AS. Extending phenotype: This phenotype involves dilation of the root plus the ascending aorta and the AAo plus the arch. Root phenotype: This refers to isolated dilation of the aortic root, a rare phenotype associated with younger age at diagnosis (<40 years), male sex, and AR (Michelena et al., 2021; Isselbacher et al., 2022). Approximately 10%–30% of BAV patients exhibit root dilation, whereas AAo dilation is observed in 55%–80% of BAV patients (Galian-Gay et al., 2019; Rodríguez-Palomares et al., 2023). Overall, the root phenotype is an independent predictor of AAo diameter growth, whereas the ascending phenotype is a stable entity with slower progression (Della Corte et al., 2013). Compared with other phenotypes, the pattern of aortopathy strongly correlates with the BAV morphotype BAV-RN (right and noncoronary cusp fusion), which is more strongly associated with and significantly higher rates of the ascending and extending phenotype. BAV-LN (left and noncoronary cusp fusion): This fusion is more frequently linked to an extended phenotype. BAV-RL (right and left cusp fusion): This fusion is more frequently linked to the root phenotype. For aortic diameter, no significant differences were observed between BAV morphotypes in AAo. However, in other regions, such as the aortic root, the diameter of BAV-RL was significantly greater than that of BAV-RN, whereas it was comparable to that of BAV-RL at the aortic root. Aortic arch: BAV-RN had a significantly larger arch diameter than did BAV-RL, with BAV-RN and BAV-LN showing similar arch diameters (Kang et al., 2013; Ruzmetov et al., 2015; Evangelista et al., 2018).

### 2.2 Genetic mechanisms of aortopathy in BAV

BAV primarily follows autosomal dominant or X-linked inheritance with familial clustering, resulting in incomplete penetrance and variable expressivity. On the one hand, BAV is often associated with complex congenital diseases such as Turner syndrome, aortic coarctation, and connective tissue disorders. On the other hand, genetic studies of BAV have revealed the interplay of several genes. For example, defects in the Jagged 1/Notch1 pathway (e.g., unbalanced translocation) can disrupt epithelial‒mesenchymal transition (EMT), cardiac neural crest cell (CNC) migration, and mesenchymal filling. Similarly, defects in GATA4/5/6, which regulate CNCs and directly contribute to aortic valve formation, play a role in BAV development (Bravo-Jaimes and Prakash, 2020; Ma et al., 2021). During cardiac development, the formation of a BAV likely occurs in three key stages: endocardial cushion formation, outflow tract (OFT) septation, and valve cushion invagination. Typically, four endocardial cushions are formed; however, during BAV development, only three cushions are created, combined with typical OFT septation, resulting in the formation of RN and LN. Abnormalities in EMT and CNC migration are thought to be the primary drivers of irregular endocardial cushion formation leading to RN or LN. Conversely, BAV-RL is hypothesized to result from abnormal or excessive fusion during embryonic OFT septation (Anderson et al., 2003; Laforest and Nemer, 2012; Soto-Navarrete et al., 2020).

The genetic correlation between BAV and thoracic aortic dilatation can be observed in the following syndromes. BAV occurs in more than 30% of Turner syndrome patients, with a significantly greater prevalence of aortic aneurysm and aortic dissection than nonsyndromic BAV patients, especially in females (Lin et al., 2021; Galian-Gay and Rodriguez-Palomares, 2022; Thunström et al., 2023). Patients with Loeys–Dietz syndrome, which involves pathogenic mutations in TGFBR1/2, often experience proximal aortic dilation and dissection at a younger age and have smaller diameters compared to those with non-complex BAVs (Loeys et al., 2005; Andelfinger et al., 2016). Marfan syndrome, characterized by connective tissue abnormalities, has an earlier onset and larger aortic aneurysms than in nonsyndromic BAV patients, particularly those involving root dilation (Sherrah et al., 2016; Yassine et al., 2017).

In a meta-analysis, the prevalence of aortic dilation among relatives of patients with BAV was found to be 29.2%, which is far higher than the 9.6% reported in relatives of patients with TAV in multicentre cohort studies (Galian-Gay et al., 2019; Bray et al., 2023). Even among relatives of patients with BAV with TAV, the overall incidence of aortic dilation was greater at approximately 10%, with a root phenotype of 3% and an ascending phenotype of 7% (Bray et al., 2023). A large-scale population-based study demonstrated that the risk of diagnosed aortic dilation in first-degree relatives of patients with BAV was 6.88 times greater and that the risk of aortic dissection was 3.63 times greater than that in the general population, which further supports a genetic association of aortopathy with BAV (Glotzbach et al., 2023). Asymptomatic thoracic aortic dilation observed in children or young adults can validate the genetic susceptibility of BAV-associated aortic dilation (Yang et al., 2020).

In addition, the genetic contributors to aortopathy associated with BAV likely involve the combined effects of abnormalities in aortic dilation-related genes, which are categorized as follows: 1. Extracellular matrix-related genes including proteoglycan-related genes (BGN, ACAN), collagen-related genes (COL1A1, COL1A2, and COL3A1), elastin-related genes (EFEMP2, ELN, FBN1, and FBN2), etc. 2. Vascular smooth muscle-related genes such as contractile protein-related genes (ACTA2), myosin-related genes (MYH11), smooth muscle-related genes (FOXE3, MAT2A, MYLK, and PRKG1), etc. 3. TGF-B signalling pathway genes such as FBN1, NOTCH1, SKI, SLC2A10, SMAD2, SMAD3, SMAD4, TGFB2, TGFB3, TGFBR1, TGFBR2, etc. (Milewicz et al., 2008; Yassine et al., 2017; Du et al., 2021).

### 2.3 Hemodynamic factors

Hemodynamic analyses using 3D or 4D MRI have revealed that long-term valvular abnormity leads to hemodynamic disturbances and altered aortic flow patterns. These include increased eccentric flow, flow velocity, eccentric jets, altered flow patterns, helical flow, systolic retrograde flow, viscous energy loss, and abnormal wall shear stress. As patients with BAV age, complication rates increase, with the onset of valvular dysfunction, particularly AS or moderate-to-severe AR, identified as independent predictors of aortic disease (Evangelista et al., 2018; Avila-Vanzzini et al., 2022). Over time, these disturbances cause thinning of the elastic fibres in the aortic wall, extracellular matrix (ECM) dysregulation, vascular wall remodelling, and the induction of atherosclerotic plaque formation. These processes promote aortic dilation and aneurysm generation, ultimately resulting in aortopathy (Bollache et al., 2018b).

There is no doubt that the biomechanical effects generated by hemodynamics play a crucial role in the pathological progression of aortic disease. As endothelial cells capable of sensing mechanical forces, they detect different characteristics of flow profiles and accordingly regulate vascular physiology and vascular remodeling. Although endothelial dysfunction and genetic susceptibility are significantly correlated, numerous studies indicate that pathological blood flow effects should not be overlooked. Endothelial cells have a variety of mechanical receptors, which can perceive alterations in blood flow hemodynamics and produce different biological responses. It has been demonstrated the inducible tyrosine phosphorylation of PECAM-1 and indicated that PECAM-1 and Src family kinases are involved in sensing/signal transduction of mechanical stimuli in endothelial cells (Osawa et al., 1997; Conway et al., 2013). Blood flow increases tension of PECAM-1, triggering the association of this protein with vimentin cytoskeleton (Conway et al., 2013). Tension on PECAM-1 triggers activation of a Src family kinase, resulting in ligand-independent transactivation of VEGF receptors and subsequent activation of PI3K, endothelial NOS, and production of nitric oxide (NO) to induce vasodilation (Fleming et al., 2005; Baeyens et al., 2016). Especially, the WSS-induced endothelial dysfunction leads to a dose-dependent secretion of NO and prostacyclin, which relaxes smooth muscle and reduces vascular tone, disrupting the homeostasis of hemodynamic mechanisms. This also induces the abnormal dose-dependent expression of the transcription factor Krüppel-like factor 2/4, increasing the expression of growth factors, oxidative elements, vasoconstrictors, acute inflammatory mediators, and proteolytic enzymes, exacerbating acute inflammation. The persistence of this process ultimately leads to arterial remodeling (Nigro et al., 2011; Baeyens et al., 2016; He et al., 2019). Similarly, in atherosclerosis, endothelial-to-mesenchymal transition in response to hemodynamic-induced vascular remodeling leads to a reduction in the expression of endothelial-specific molecules, including VE-cadherin and PECAM-1, in order to balance the effects of pro-inflammatory factors (Chen et al., 2015). By modulating the extracellular matrix, it facilitates the progression of inflammation, such as the matricellular protein CCN1, which persistently promotes atherogenesis (Hsu et al., 2019; Chen et al., 2024). Furthermore, endothelial dysfunction can lead to dysregulate NO expression, aberrant NOTCH signaling, and increased calcium deposition, which collectively contribute to the development of calcific aortic valve disease. This calcification impairs valve function, resulting in AS or AR, which further exacerbates hemodynamic disturbances (Antequera-González et al., 2020). Recent studies also highlight the role of hemodynamic forces in inducing epigenetic modifications, with endothelial cells producing Mechano-microRNAs in response to WSS exposure, thereby intensifying arterial remodeling and promoting inflammatory responses (Rashad et al., 2020).

In response to altered hemodynamics, such as high or low WSS, cells can activate various signaling pathways (e.g., NOTCH and TGF-β), leading to changes in gene expression that influence vascular function (Milewicz et al., 2017; Baek et al., 2022). A basic research has found that changes in hemodynamics and increased WSS mediate the Notch-ephrinb2 pathway, promoting the formation of new arterial rings and restoring microcirculation (Baek et al., 2022). Numerous studies have shown that elevated WSS is closely associated with downstream molecules of TGF-β, such as MMPs, SMAD2/3, and SM22 alpha protein, indicating that hemodynamic signals activate the TGF-β signaling pathway, leading to changes in vascular elastin molecules and resulting in vascular remodeling (Guzzardi et al., 2015; Kunnen et al., 2017; Sophocleous et al., 2018).

## 3 Clinical management of BAV

The surgical treatment of aortopathy associated with BAV focuses on addressing the underlying causes of aortic valve dysfunction. Procedures include surgical aortic valve replacement (SAVR), transcatheter aortic valve replacement (TAVR) and BAV repair (Fraser et al., 1994; Ehrlich et al., 2020; Halim et al., 2020; Kalogeropoulos et al., 2022; Bavaria et al., 2024). For patients with aortic aneurysms, aortic dissection, aortic rupture, or those at high risk for cardiovascular events such as those mentioned previously, surgical or prophylactic interventions are recommended. These include ascending aorta replacement (AAR), aortic root replacement (ARR), valve-sparing aortic root replacement (VSARR), and Bentall root replacement (Sievers et al., 2014; Beckmann et al., 2020; Mokashi et al., 2022; Levine et al., 2023).

Recommendations from the 2022 Guidelines: Class I Recommendation: Surgery is advised for BAVs with an aortic root or ascending aortic diameter ≥55 mm, or both. Class IIa Recommendation: It is reasonable for experienced surgeons within a multidisciplinary aortic team to perform surgery for patients with aortic root or ascending aortic cross-sectional area-to-height ratios ≥10 cm^2^/m. For BAVs and aortic root or AAo diameters of 50–54 mm, along with additional risk factors for dissection, surgery is also considered reasonable. Concurrent AAR or ARR is advised in patients who undergo surgical aortic valve repair or replacement when the diameter reaches ≥45 mm. Class IIb Recommendation: For BAVs and aortic root or ascending aortic diameters of 50–54 mm without additional risk factors, surgery may be considered reasonable (Masri et al., 2017; Borger et al., 2018; Otto et al., 2021; Isselbacher et al., 2022).

Partial arch replacement is the most common surgical technique, with a lower incidence of concomitant cardiac procedures. The VSARR is increasingly used for BAVs with root dilation (Kayatta et al., 2019; Nguyen et al., 2021; Levine et al., 2023). Most patients do not reach the elective repair threshold of 55 mm, even in the presence of aortic dissection. Therefore, prophylactic aortic surgery should be considered on the basis of a comprehensive assessment. Given that familial thoracic aortic aneurysm patients often present dissection at younger ages with poorer outcomes and a greater likelihood of reintervention, prophylactic surgery is necessary when the maximum diameter of the root or ascending aorta reaches ≥50 mm (Brownstein et al., 2018; Saeyeldin et al., 2019). In a retrospective study, more than two-thirds of patients with BAV underwent prophylactic aortic root replacement during the perioperative period of aortic dissection, whereas this was the case for only 19% of patients with TAV (Kreibich et al., 2020).

The choice of aortic surgical intervention varies across different valve types. BAV type 2 valves are significantly more likely to require AAR at threshold diameters. BAV-RL cusp fusion and valvular dysfunction are associated with increased frequencies of AAR. Root interventions (Bentall, David, Yacoub, or other remodelling procedures) are more common in BAV-RL patients with AR than in those with associated AS (Sievers et al., 2014).

Age is an independent predictor of aortic pathology in patients with BAV and plays a crucial role in surgical decision-making, especially in paediatric patients. For paediatric patients with BAV, the treatment of aortic dilation is individualized. In children, the 2024 scientific statement suggests standardizing the measurement of body surface area-adjusted z scores to define aortic root and ascending aortic dilation, where dilation is classified as a z score >2 SD (Devereux et al., 2012; Grattan et al., 2020; Morris et al., 2024). Isolated dilation of the aortic root or ascending aorta is rarely an indication for surgery in children and adolescents (Niaz et al., 2020). The statement recommends tailoring surgical thresholds for the root or ascending aorta on the basis of genetic mutations and high-risk profiles (Hiratzka et al., 2010; Morris et al., 2024). For patients with heritable thoracic aortic disease (HTAD), elective surgery for root aneurysms should involve replacing the entire ascending aorta along with the root, and selective proximal arch replacement (partial arch replacement) may offer protection when performed by experienced centres (Malaisrie et al., 2015; Preventza et al., 2017). Total arch replacement is not recommended for isolated root aneurysms undergoing elective repair (Regalado et al., 2018). For paediatric patients with BAV, medical conservative treatment aims to slow the progression of aortic dilation. A retrospective study demonstrated that, compared with no treatment, treatment with losartan or atenolol resulted in slower aortic growth. Additionally, guidelines emphasize the management of activity and exercise in paediatric patients (Baleilevuka-Hart et al., 2020; Niaz et al., 2020; Flyer et al., 2021).

## 4 Hemodynamic characteristics in BAV patients

### 4.1 Wall shear stress

WSS is a widely studied hemodynamic parameter in BAV patients. The congenital anomaly of the BAV is thought to increase the WSS, exerting a vicious force on the aortic wall and increasing regional abnormalities. We summarized the distribution of abnormal WSS in different aortic regions on the basis of recent studies (Table 1). However, the BAV morphology, aortic diameter, and valvular dysfunction are all factors influencing hemodynamics. In patients with BAV, the presence of aortic dilation influences the WSS distribution, with greater wall stress at the aortic root and significant increases on the inner and right sides of the AAo and the outer and left sides of the arch, whereas those without dilation present significantly elevated WSS throughout most of the inner AAo and the entire arch (Dux-Santoy et al., 2020; Gomez et al., 2020). We hypothesize that long-term WSS abnormalities lead to compensatory aortic dilation to mitigate the effects of WSS.

**TABLE 1:** Regional distribution of abnormal WSS.

	Dilated/Non-dilated	WSS			
		Magnitude WSS	Axial WSS	Circumferential WSS	WSS angle
1 (Minderhoud et al., 2024)	Non-dilated	Inner root Outer root Outer proximal AAO Outer distal AAO	Entire aorta Inner root Outer root Inner proximal AAO Inner distal AAO	Entire aorta Inner root Outer root Inner proximal AAO Outer proximal AAO Inner distal AAO Outer distal AAO	Entire aorta Inner root Outer root Inner proximal AAO Outer proximal AAO Inner distal AAO Outer distal AAO
2 (Gilles et al., 2022)	Non-dilated	-	proximal AAO	proximal AAO Entire AAO	proximal AAO Entire AAO
3 (Bollache et al., 2018b)	Dilated	Greater curvature aorta Posterior wall aorta			
4 (Geeraert et al., 2021b)	Non-dilated and Dilated	LVOT SOV	SOV	LVOT SOV MAA AA1	NA
	Non-dilated	LVOT* MAA* AA1*	LVOT* AA1*	-	NA
5 (Dux-Santoy et al., 2020)	Non-dilated	Inner AAO Right AAO Outer AAO Entire Arch			NA
	Dilated	Right AAO Outer AAO Outer Arch Left Arch Inner distal Arch			NA
6 (Rodríguez-Palomares et al., 2018)	Non-dilated& Dilated	-	Entire AAO	Entire AAO	NA
	Dilated-root	-	proximal AAO+ mid AAO+	-	NA
	Dilated-ascending	-	-	Entire AAO+	NA
7 (Dux-Santoy et al., 2019)	Non-dilated& Dilated	NA	-	Proximal arch	NA
	Non-dilated	NA	Proximal arch*	-	NA

LVOT, left ventricular outflow tract; MAA, mid-ascending aorta; SOV, sinuses of Valsalva; AA1, proximal to first aortic branch; *significant higher compare to Dilated/Non-dilated group; +significant higher compare to Dilated root/ascending group.

Recent research involving 72 BAV patients by Soulat et al. further demonstrated that elevated WSS regions are predictive of an increased aortic expansion rates (Soulat et al., 2022). A prospective study demonstrated the critical role of the WSS and its circumferential component in predicting progressive aortic dilation (Guala et al., 2022). In addition to the WSS, derived parameters such as axial, circumferential, and magnitude WSS, and the oscillatory shear index (OSI), have shown wide value in hemodynamic analyses. Aortic diameter growth in patients with BAV is strongly correlated with the circumferential WSS and WSS angle, whereas the axial WSS and OSI behave in opposite directions, resulting in a decrease in areas of aortic expansion (Table 2) (Guala et al., 2022; Minderhoud et al., 2022; Soulat et al., 2022). Table 1 highlights that despite varying regional segmentation methods, WSS abnormalities and derived parameters are predominantly concentrated in the aortic root and proximal ascending aorta. JosΩ Fernando and Patrick Geeraert et al. reported that the circumferential WSS and WSS angle are strongly correlated with the root and ascending phenotypes, whereas the magnitude of the WSS and axial WSS are more strongly associated with the root phenotype (Rodríguez-Palomares et al., 2018; Geeraert et al., 2021b). Notably, WSS-derived parameters at the aortic arch level are less significant than those in the AAo are, particularly in the distal arch, where the WSS tends to decrease without notable differences compared with that of controls (Dux-Santoy et al., 2019).

**TABLE 2:** Abnormal hemodynamic parameters in ascending aorta.

	BAV	Type of BAV		Valve dysfunction		Ascending aortic diameter		Associated with dilation
		RN	RL	As	AR	Dilated	Not-dilated	
Flow angle	^b^(Rodríguez-Palomares et al., 2018)	^d^(Bissell et al., 2013; Rodríguez-Palomares et al., 2018) NO(Dux-Santoy et al., 2019)	^c^(Mahadevia et al., 2014) NO (Dux-Santoy et al., 2019)	^c^(Bissell et al., 2013)		^d^(Rodríguez-Palomares et al., 2018) NO (Dux-Santoy et al., 2019)		^b^(Elbaz et al., 2019)
Normalized displacement	^b^(Rodríguez-Palomares et al., 2018)	^c^(Mahadevia et al., 2014) NO (Dux-Santoy et al., 2019)	^d^(Rodríguez-Palomares et al., 2018) NO (Dux-Santoy et al., 2019)			^d^(Rodríguez-Palomares et al., 2018; Dux-Santoy et al., 2019)		^b^(Elbaz et al., 2019)
Rotational flow	^b^(Rodríguez-Palomares et al., 2018)	^d^(Bissell et al., 2013; Rodríguez-Palomares et al., 2018; Dux-Santoy et al., 2019)		^d^(Bissell et al., 2013)		^d^(Rodríguez-Palomares et al., 2018; Dux-Santoy et al., 2019)		
Retrograde flow	^b^(Rodríguez-Palomares et al., 2018; Geeraert et al., 2021b)	^c^(Rodríguez-Palomares et al., 2018; Dux-Santoy et al., 2019)				^d^(Rodríguez-Palomares et al., 2018; Dux-Santoy et al., 2019)		^b^(Rodríguez-Palomares et al., 2018)
Peak velocity	^b^(Rodríguez-Palomares et al., 2018; Geeraert et al., 2021b)	^c^(Bissell et al., 2013; Dux-Santoy et al., 2019) ^d^(Rodríguez-Palomares et al., 2018)		^d^(Bissell et al., 2013)		^c^(Dux-Santoy et al., 2019) ^d^(Rodríguez-Palomares et al., 2018)		
TKE/KE				^c^(Elbaz et al., 2019) ^d^(Binter et al., 2017)	^d^(Elbaz et al., 2019)			
Wear shear stress		^c^(Mahadevia et al., 2014) ^d^(Bissell et al., 2013)		^d^(Bissell et al., 2013)				^b^(Soulat et al., 2022)
Magnitude	a (Minderhoud et al., 2022)						^d^(Geeraert et al., 2021b)	^f^(Geeraert et al., 2021b)
Axial	^e^ (Minderhoud et al., 2022) ^f^ (Rodríguez-Palomares et al., 2018)		^c^(Dux-Santoy et al., 2019) ^d^(Rodríguez-Palomares et al., 2018)				^c^(Geeraert et al., 2021b) ^d^(Rodríguez-Palomares et al., 2018; Dux-Santoy et al., 2019)	
Circumferential	^b^(Rodríguez-Palomares et al., 2018; Geeraert et al., 2021b; Minderhoud et al., 2022)	^d^(Rodríguez-Palomares et al., 2018; Dux-Santoy et al., 2019) NO (Bissell et al., 2013)	NO (Bissell et al., 2013)	^d^(Bissell et al., 2013)		^c^(Geeraert et al., 2021b) ^d^(Rodríguez-Palomares et al., 2018; Dux-Santoy et al., 2019)		^b^(Rodríguez-Palomares et al., 2018)

a : higher vs. health volunteers/not mentioned P (<0.05).

b : significant higher vs. health volunteers, or positively association with dilation.

c : higher vs. subgroup counterpart/severe stenosis vs. no or mild or moderate stenosis/insufficiency vs. no insufficiency/dilated vs. non-dilated.

d : significant higher vs. subgroup counterpart/severe stenosis vs. no or mild or moderate stenosis/insufficiency vs. no or mild or moderate insufficiency/dilated vs. non-dilated.

e : lower vs. health volunteers/not mentioned P(<0.05).

f : significant higher vs. health volunteers, or negatively association with dilation.

Age is an independent factor for BAV-associated aortic events (Tzemos et al., 2008; Thompson et al., 2023). Following the initial diagnosis, temporal changes in the WSS are factors to be considered. The systolic peak WSS and the relative area of elevated WSS showed no significant changes over several scans, and patients with BAV with a low aortic growth rate presented favourable long-term stability across multiple imaging sessions (Maroun et al., 2024). In contrast, a 3-year follow-up study of young patients with BAV demonstrated increasing magnitude, axial, and circumferential WSS over time, regardless of aortic growth. Compared with that of controls, the circumferential WSS was greater across all ascending aorta regions, with the most significant WSS abnormalities occurring in the outer proximal AAo (Minderhoud et al., 2024).

In vascular biology studies, elevated WSS levels have been linked to increased nitric oxide production by endothelial cells, leading to vascular dilation and subsequent structural changes in the vessel wall. Hemodynamic changes due to valvular abnormalities result in WSS inversely correlated with elastin quantity, widening interstitial spaces, thinning fibrillin fibres, and increased matrix metalloproteinases, driving extracellular matrix (ECM) remodelling and ascending aortic wall structural changes (Guzzardi et al., 2015; Bollache et al., 2018b).

### 4.2 Eccentric blood flow

Previous studies have demonstrated that eccentric blood flow (quantified by normalized displacement and flow angle) is associated with accelerated aortic growth in patients with BAV, particularly in patients with the ascending aorta dilation phenotype (Bissell et al., 2013; Hope et al., 2014; Raghav et al., 2018; Rodríguez-Palomares et al., 2018). Eccentric flow can be characterized using the parameters of normalized displacement and flow angle (Edlin et al., 2019). The velocity angle proved to be an excellent biomarker for differentiating between volunteers and patients with BAV, BAV morphotypes, and BAV phenotypes (Sotelo et al., 2022). Compared with patients with TAV with matched aortic diameters and valve function, patients with BAV exhibit significantly greater normalized displacement, which varies by valve morphology (Mahadevia et al., 2014). These variables are greater in the proximal aorta and progressively decrease in the distal AAo, indicating that the flow tends to become more symmetric in the distal AAo (Rodríguez-Palomares et al., 2018). In addition, patients with BAV with significantly eccentric flow demonstrated faster growth (Hope et al., 2014).

### 4.3 Flow patterns

Altered flow patterns in aortopathy can accelerate its progression. Helical flow, defined as localized fluid circulation along the longitudinal axis of a vessel, is a key parameter reflecting the relationship between flow velocity and vorticity. In healthy individuals, aortic blood flow follows a right-handed (clockwise) helical pattern through the ascending aorta and aortic arch (Bogren and Buonocore, 1999; von Knobelsdorff-Brenkenhoff et al., 2014). This requires the WSS and helicity index to measure the degree of helicity.

Patients with BAV exhibit increased right-handed helical flow (pathological right-handed helicity) characterized by spiral flow lines extending from the aortic root to the beginning of the aortic arch (Shan et al., 2017). In a study conducted by Patrick Geeraert and colleagues of 73 BAV patients, small helical flow regions were observed in the aortic arch and proximal descending aorta, whereas larger helical flow zones with significant reverse flow directions were detected in the AAo (Geeraert et al., 2021a). In patients with BAV with pathological right-handed helical flow, the aortic diameter increased in proportion to the severity of flow turbulence. Conversely, in patients with normal flow patterns, the aortic dimensions were similar to those in the control group (Bissell et al., 2013). The WSS angle, an indicator of helicity near the aortic wall, typically decreases in dilated aortic regions. It peaks in the medial proximal ascending aorta, indicating maximal helical flow at the aortic wall in this region, which is consistent with the previously described zones of flow disturbance (Minderhoud et al., 2024).

### 4.4 Viscous energy loss and kinetic energy

Viscous energy loss (EL) represents the energy dissipated within blood flow due to frictional forces and, in the ascending aorta, can serve as an indicator of left ventricular afterload and cardiac events (Geeraert et al., 2021a). In patients with BAV, both the systolic EL and the EL index in the aorta are significantly elevated compared with those in healthy volunteers, which correlates with increased aortic diameters at the mid AAo and proximal AAo to the first aortic branch planes (Geeraert et al., 2021b). Kinetic energy (KE) quantifies flow velocity and energy dynamics within the aorta. Some studies have reported minimal differences in KE at regions of the aortic root and AAo, whereas other investigations have failed to observe statistically significant changes (Elbaz et al., 2019; Geeraert et al., 2021a). KE emphasizes the overall energy of blood flow, whereas turbulent kinetic energy (TKE) focuses on the energy fluctuations caused by turbulent blood flow. Therefore, the TKE might better represent the hemodynamics observed in BAV patients (Ferrari and Obrist, 2024). Both EL and TKE show substantial increases in BAV patients, particularly within the AAo (Dux-Santoy et al., 2019; Elbaz et al., 2019).

### 4.5 BAV morphotypes association with aortopathy

Valve morphology is a key determinant of the severity of aortic dilation, with distinct hemodynamic patterns observed among different BAV phenotypes (Bissell et al., 2013; Rodríguez-Palomares et al., 2018). Different cusp fusion patterns (BAV-RL and BAV-RN) create distinct regional variations in WSS patterns. In BAV-RL patients, the WSS along the outer curvature of the aortic root and ascending aorta is significantly elevated by 9%–34% compared with that in controls, whereas in BAV-RN patients, the WSS increases by 30% in the distal aorta (van Ooij et al., 2017; Rodríguez-Palomares et al., 2018; Dux-Santoy et al., 2020). Compared with BAV-RL, BAV-RN results in more severe flow abnormalities characterized by increased AAo dilation, exacerbated eccentric flow, extensive regions of significantly elevated normalized displacement localized at the proximal AAo, and intensified rotational flow in the proximal aortic arch (Mahadevia et al., 2014; Shan et al., 2017). Regarding flow patterns, both the RN and RL types are associated with a predominance of right-handed helical flow; however, the RL type results in a greater proportion of normal flow and an absence of left-handed helical flow (Bissell et al., 2013). This may explain the significant differences in hemodynamic parameters between BAV-RN and BAV-RL observed in the aforementioned studies (Table 2).

Many studies now focus on the morphological characteristics of the valve itself, particularly the commissural angle. BAVs with highly asymmetric commissures (120° commissural angle) exhibit a reduced aortic orifice area during systolic peak flow, with jet streams impacting the right posterior wall of the proximal ascending aorta, leading to elevated WSS. In contrast, more symmetric commissures (180° commissural angle) diminish jet impacts on the posterior wall, resulting in linearly reduced stress and strain levels on the unfused noncoronary cusp (Murphy et al., 2017; Yeats et al., 2024).

### 4.6 BAV dysfunction association with aortic pathology

Numerous studies have shown that BAV functional phenotypes (e.g., aortic stenosis and aortic regurgitation) are significantly associated with the severity and progression of aortopathy (Girdauskas et al., 2017). In AS patients, the WSS, viscous EL, TKE, and retrograde flow in the AAo are all elevated and correlate with the severity of stenosis, which is primarily localized to the outflow tract and proximal AAo (Dyverfeldt et al., 2013; Binter et al., 2017; Christian et al., 2017; Elbaz et al., 2019; Geeraert et al., 2021b). Hemodynamic studies on aortic valve dysfunction have revealed that all degrees of AS lead to localized WSS increases in both BAV and TAV patients. Compared with healthy individuals, mild AS significantly increases the WSS in the lateral region of the proximal AAo, which contrasts with TAV-associated aortic dilation accompanied by mild AS. Moderate to severe AS results in a significant increase in WSS across nearly the entire AAo and aortic root, irrespective of the valve phenotype (van Ooij et al., 2017). Yan Shan et al. further confirmed that, regardless of the presence of valvular dysfunction, the location of the peak WSS in the BAV-RL aligns with the valve type and tends to converge in the middle and distal regions of the AAo (Shan et al., 2017; 2019). Thus, we believe that an elevated WSS appears to be a characteristic mechanism underlying aortic dilation in patients with AS, overriding the influence of aortic valve morphology.

In contrast, severe AR is characterized by localized, noneccentric WSS increases that are positively correlated with stroke volume (Shan et al., 2017). A recent cross-sectional study by Elizabeth K. Weiss indicated that in BAV patients, with increasing severity of AR, there is a time-dependent increase in retrograde flow in the AAo, particularly during diastole. Moreover, they demonstrated that AAo dilation is independently associated with AR in BAV patients (Weiss et al., 2023; Fujiwara et al., 2024). The oscillatory shear index (OSI) appears to be the sole hemodynamic parameter capable of identifying abnormalities in BAV patients with AR. OSI elevation predominantly occurs in the outer mid-to-distal AAo and aortic root, but it does not appear to be associated with the region of maximal dilation in patients with BAV. An elevated OSI may result from the combined effects of inherent regurgitant flow and increased stroke volume associated with AR. Moreover, the greater the severity of AR is, the higher the OSI value. Therefore, OSI elevation may represent a characteristic mechanism of aortic valve regurgitation but is weakly associated with the region of aortic dilation (Dux-Santoy et al., 2020; Trenti et al., 2024).

### 4.7 Hemodynamic characteristics of paediatric BAV patients

Compared with adults, paediatric BAV patients exhibit greater variability in age, weight, and aortic dimensions, along with distinct hemodynamic parameters. Similar to adults, the severity of valvular dysfunction and the BAV-RN phenotype can serve as independent factors for AAo dilation in paediatric or young patients (Grattan et al., 2020). Significant correlations exist between age and peak systolic velocity and WSS, whereas retrospective studies on paediatric BAV patients have shown that the WSS direction and velocity direction are correlated with AAo z scores rather than with the WSS magnitude as in adults (Allen et al., 2015; van Ooij et al., 2016; Fujiwara et al., 2024). A longitudinal study with a follow-up of 3.4 years confirmed that peak velocity in the AAo serves as a predictor of aortic dilation in young and paediatric BAV patients (Rose et al., 2019). In particular, in children with valvular dysfunction, the peak velocity is significantly increased across all aortic segments (Stefek et al., 2019). Similar to adults, the correlation between hemodynamic parameters and aortic diameter varies across anatomical planes in paediatric BAV patients. For example, the diameters of the aortic root and the sino tubular junction (STJ) are significantly associated with vorticity and KE but not with peak velocity (Henry et al., 2023). Peak velocity and WSS are greater in the AAo region than in the other regions (Allen et al., 2015; Stefek et al., 2019; Fujiwara et al., 2024). Overall, compared with that in adults, research on paediatric BAV patients remains limited, with fewer hemodynamic parameters studied and substantial variability in aortic dimensions hindering the establishment of reliable reference data.

## 5 Hemodynamic characteristics of postsurgical BAV patients

Currently, significant hemodynamic changes are observed in patients prior to surgery. We also reviewed their hemodynamic characteristics after surgery.

### 5.1 Aortic valve replacement (AVR) and aortic root replacement (ARR)

Multiple studies have shown that post-AVR peak flow and WSS remain elevated in patients compared with healthy individuals, and there are also significant differences compared with native valves (von Knobelsdorff-Brenkenhoff et al., 2014; Collins et al., 2015; Farag et al., 2019). A study of the postoperative outcomes of various valve types in AVR revealed that all valve types demonstrated eccentric flow, which was primarily concentrated on the right-anterior aortic wall (von Knobelsdorff-Brenkenhoff et al., 2014). Hemodynamic studies comparing TAVR and SAVR have suggested that post-TAVR, the eccentricity and displacement of blood flow are altered in the middle and distal segments of the ascending aorta, whereas SAVR primarily affects the distal AAo (Farag et al., 2019). However, this study did not clarify the extent of hemodynamic improvement postoperatively compared with the preoperative state. In a 4D flow study of AS patients, comparisons of hemodynamic changes before and after AVR revealed a 30% increase in flow rate and a reduction in the AAo flow angle from 39° to 25° (Kamada et al., 2020). Yuki Yoshioka and colleagues suggested that surgeries such as AVR and AAR reduce eccentric jets, thereby lowering WSS (Bollache et al., 2018a; Yoshioka et al., 2023). Recently, an *in-silico* study demonstrated that post-AVR hemodynamics exhibit weaker, less eccentric jets and increased OSI. Significant reductions are observed in flow helicity, time-averaged WSS and WSS divergence in localized regions of the proximal AAo (Bailoor et al., 2024).

Eric J. Keller et al. reported that in 10 patients who underwent the standard Bentall procedure, the abnormal hemodynamics observed before aortic root replacement (ARR) were significantly reduced. The helicity and vorticity levels significantly decreased, restoring flow patterns similar to those in healthy individuals (Keller et al., 2016). ARR reduces the local WSS, whereas the proximal WSS decreases after ARR, the graft distal WSS increases, and no significant temporal changes are observed (Bollache et al., 2018a). As WSS is a primary flow parameter for aortic dilation in BAVs, we hypothesize that changes in postoperative WSS could predict alterations in aortic diameter. However, large-scale studies on this correlation are still lacking. Altered flow patterns may reflect improved aortopathy and normalization of the aortic flow pattern postsurgery.

### 5.2 Valve-sparing aortic root replacement (VSARR)

In a cohort primarily comprising TAV patients with approximately 3 years of follow-up, VSARR was associated with significantly greater EL in the proximal aorta than was the healthy group. The maximum flow velocity in the distal AAo was significantly elevated relative to that in healthy controls. However, parameters such as pulse wave velocity, WSS, and flow patterns (eccentricity, vorticity, and helicity) did not significantly differ (Cvitkovic et al., 2022). In a mixed TAV and BAV population study, VSARR patients retained valves and exhibited lower flow eccentricity with no AAo wall jetting. However, helical flow remained significantly greater than that in healthy individuals, and the systolic peak velocity was also elevated, which was different from previous findings. In studies involving only TAV patients, the flow velocity and WSS in the ascending and descending aorta were significantly greater, particularly in distal graft regions where the axial WSS was notably greater (Collins et al., 2015; Gaudino et al., 2019; Oechtering et al., 2020). TAV patients cannot fully represent BAV patients who undergo AVR (Kaiser et al., 2022). Stephens et al. reported that among 19 BAV patients who underwent VSARR, most exhibited prominent right-handed or left-right helical flow in the AAo, although the study did not specify whether these were pathological patterns (Stephens et al., 2015). After VSARR, BAV patients continue to exhibit greater asymmetric WSS throughout the entire AAo than healthy individuals do (Stephens et al., 2015). Post-VSARR, BAV patients indeed show significant hemodynamic improvement and clinical outcomes, indicating long-term survival rates and reoperation rates comparable to those of the Bentall procedure (Kalra et al., 2021; Bethancourt et al., 2022; Zuo et al., 2023). However, whether retained abnormal leaflets and age-related valve dysfunction could further impact the postoperative aorta, as well as unoperated native arteries, requires further follow-up studies.

Overall, there is significant improvement in postoperative hemodynamics following AVR/ARR, but eccentric blood flow and localized WSS remain present postoperatively. In the VSARR data reviewed, the TAV cohort was able to return to normal hemodynamic characteristics. Meanwhile, the mixed cohort or solely BAV cohort exhibited less eccentric blood flow, with only the flow patterns remaining abnormal. Of course, this result is limited by factors such as surgical selection bias in the population and the insufficient postoperative hemodynamic data for VSARR. However, these postoperative hemodynamic data will also provide valuable references for surgical approach selection and postoperative prognostic evaluation.

## 6 Limitations and future directions

The relationship between aortic dilation and hemodynamics in BAV patients is complex. However, in current studies, most patients do not have documented genetic characteristics, making it difficult to distinguish the overlapping contributions of hemodynamic and genetic factors. Aortic dilation may result from hemodynamic alterations, or alternatively, it could stem from genetic factors that lead to dilation, subsequently causing hemodynamic abnormalities. Recently, via fluid‒structure interaction simulations, it has become possible to isolate the impact of valve morphology on hemodynamics in patients with aortic disease. This study may isolate the contribution of valve-related genetic abnormalities to the aortic dilation (Kaiser et al., 2022). Current international guidelines and criteria for surgical intervention now incorporate genetic considerations, which aid in the early identification of complications and prognosis in BAV patients. However, the genetic factors influencing complications and surgical outcomes at this stage remain unclear (Michelena et al., 2021; Isselbacher et al., 2022).

Although there has been extensive preoperative 4D flow hemodynamic research on BAV patients, the focus has largely been limited to parameters such as the WSS and peak velocity. The data types are inconsistent, and even when similar data types are used, their representation varies, lacking standardized formats (Cave et al., 2021). Comprehensive large-scale, multicentre cohort studies are still lacking, as are outcome prediction models. The potential value of hemodynamic parameters in aortopathy has yet to be fully explored. The assessment of pediatric BAV patients still faces significant challenges in the future. On one hand, there are difficulties in standardizing evaluations due to age, size and genetic variability. On the other hand, there is also a lack of hemodynamic data regarding long-term outcomes after different surgical procedures for pediatric BAV patients. There is also a lack of clinical research data comparing the hemodynamics of children and adults.

Postoperative 4D flow hemodynamic studies are limited in quantity and diversity, with most failing to differentiate between bicuspid and tricuspid valve patients. There is a lack of clarity regarding whether hemodynamic parameters improve postoperatively in BAV patients, what constitutes improvement criteria, and what the definitive prognostic outcomes are (Collins et al., 2015; Gaudino et al., 2019; Oechtering et al., 2020; Cvitkovic et al., 2022). Hemodynamic assessments for evaluating prognostic correlations in postoperative patients also remain underexplored. There is a lack of postoperative hemodynamic data research focused on specific surgical techniques particularly AAR.

The integration of machine learning and AI has advanced the automated evaluation of hemodynamic patterns. However, there remains a need for further development in the qualitative and quantitative analysis of relevant parameters, as well as in the breadth of data types. Prognostic assessments in this domain also remain underdeveloped (Haji-Valizadeh et al., 2021; Peper et al., 2022; Kim et al., 2023). Developing predictive models based on future advancements in hemodynamics holds significant importance not only for predicting outcomes in BAV patients but also for those with other aortic-related diseases.
